# Deep Learning-Enhanced Portable Chemiluminescence Biosensor: 3D-Printed, Smartphone-Integrated Platform for Glucose Detection

**DOI:** 10.3390/bioengineering12020119

**Published:** 2025-01-27

**Authors:** Chirag M. Singhal, Vani Kaushik, Abhijeet Awasthi, Jitendra B. Zalke, Sangeeta Palekar, Prakash Rewatkar, Sanjeet Kumar Srivastava, Madhusudan B. Kulkarni, Manish L. Bhaiyya

**Affiliations:** 1 Department of Electronics Engineering (Biomedical Engineering), Ramdeobaba University, Nagpur 440013, Indiaawasthiar_1@rknec.edu (A.A.); 2 Department of Electronics Engineering, Ramdeobaba University, Nagpur 440013, India; 3 Bio Spectronics Private Limited, Nagpur 440013, India; 4 Department of Mechanical Engineering, Technion, Israel Institute of Technology, Haifa 3200003, Israel; 5 Department of Electrical & Electronics Engineering, Birla Institute of Technology & Science Pilani, Hyderabad Campus, Hyderabad 500078, India; 6 Department of Electronics and Communication Engineering, Manipal Institute of Technology, Manipal Academy of Higher Education (MAHE), Manipal 576104, India; 7 Department of Chemical Engineering and the Russell Berrie Nanotechnology Institute, Technion, Israel Institute of Technology, Haifa 3200003, Israel

**Keywords:** deep learning, portable biosensor, paper-based WPµ-pad, chemiluminescence detection, smartphone integration, 3D-printed sensor, bio-analyte detection, point-of-care (PoC) diagnostics

## Abstract

A novel, portable chemiluminescence (CL) sensing platform powered by deep learning and smartphone integration has been developed for cost-effective and selective glucose detection. This platform features low-cost, wax-printed micro-pads (WPµ-pads) on paper-based substrates used to construct a miniaturized CL sensor. A 3D-printed black box serves as a compact WPµ-pad sensing chamber, replacing traditional bulky equipment, such as charge coupled device (CCD) cameras and optical sensors. Smartphone integration enables a seamless and user-friendly diagnostic experience, making this platform highly suitable for point-of-care (PoC) applications. Deep learning models significantly enhance the platform’s performance, offering superior accuracy and efficiency in CL image analysis. A dataset of 600 experimental CL images was utilized, out of which 80% were used for model training, with 20% of the images reserved for testing. Comparative analysis was conducted using multiple deep learning models, including Random Forest, the Support Vector Machine (SVM), InceptionV3, VGG16, and ResNet-50, to identify the optimal architecture for accurate glucose detection. The CL sensor demonstrates a linear detection range of 10–1000 µM, with a low detection limit of 8.68 µM. Extensive evaluations confirmed its stability, repeatability, and reliability under real-world conditions. This deep learning-powered platform not only improves the accuracy of analyte detection, but also democratizes access to advanced diagnostics through cost-effective and portable technology. This work paves the way for next-generation biosensing, offering transformative potential in healthcare and other domains requiring rapid and reliable analyte detection.

## 1. Introduction

The need for rapid, accurate, and portable diagnostic tools has grown significantly with the increasing prevalence of diseases requiring timely detection and management [1]. Biosensors, as analytical devices that integrate a biological recognition element with a transducer, have emerged as transformative tools for detecting a wide range of analytes, including glucose, urea, creatinine, and disease biomarkers [2]. The conventional biosensing tools, such as electrochemical [3,4], optical [5,6], piezoelectric [7,8], and opto-acoustics biosensors [9,10], have proven effective in clinical diagnostics [11], environmental monitoring [12], and food safety [13]. However, these methods often face challenges related to cost, complexity, and scalability, particularly in resource-limited settings. The field of biosensing has witnessed a paradigm shift over the last few decades, transitioning from the traditional laboratory-based methods to portable, point-of-care (PoC) devices [14,15,16,17,18,19]. Biosensors are analytical tools that combine biological recognition elements with transducers to detect specific analytes, offering applications in healthcare, environmental monitoring, food safety, and more [20,21,22].

Among these, the development of portable biosensors is especially significant for resource-limited settings, where access to centralized laboratories is often constrained [23,24,25]. Chemiluminescence (CL)- and electrochemiluminescence (ECL)-based biosensors have emerged as a promising alternative due to their high sensitivity, broad dynamic range, and minimal background interference [26,27]. Chemiluminescence, a phenomenon where light is emitted as a result of a chemical reaction, has gained prominence in biosensing due to its exceptional sensitivity and specificity. Unlike fluorescence, chemiluminescence does not require an external light source, eliminating the background noise and reducing instrumentation complexity. This technique is widely employed in immunoassays, enzyme assays, and nucleic acid detection [28,29]. The core advantage of the CL method lies in its ability to produce strong, quantifiable signals even at low analyte concentrations. Reactions involving luminol, acridinium esters, or peroxyoxalates are commonly used in chemiluminescent biosensors, resulting in exceptional sensitivity and specificity in detecting bio-analytes, including proteins, nucleic acids, and small molecules [30,31]. Despite these benefits, the traditional chemiluminescence systems face significant barriers to adoption in PoC applications. The conventional setups rely on expensive photomultiplier tubes (PMTs) or CCD cameras for signal detection, which are impractical for portable and affordable devices. Additionally, the lack of real-time, automated signal analysis further limits their usability [32].

Traditionally, the quantitative measurement of glucose in various samples has relied on large-scale CL devices [33]. These conventional CL systems, while effective in laboratory settings, present significant limitations. They typically require large volumes of reagents and samples, involve multiple manual operational steps, and necessitate lengthy analysis processes. Additionally, their dependence on complex and bulky peripheral equipment makes them impractical for PoC or on-site glucose testing applications [34]. To address these challenges, microchip-based microfluidic chemiluminescent devices have been developed, offering potential improvements in glucose sensing [35,36]. These systems integrate microfluidic technology with CL detection to minimize reagent and sample use, streamline the workflows, and reduce the overall size of the sensing platforms. However, despite these advancements, the existing microfluidic CL systems still face notable drawbacks.

Two significant issues lie in the complexity and cost of chip fabrication. Many microfluidic CL devices require intricate manufacturing processes and the use of expensive substrate materials, making them less feasible for widespread or cost-effective production. Additionally, these devices often rely on costly peripheral equipment. For example, solution handling typically involves valving and pumping systems, while specialized tubing is needed to connect the microchannels with these components [37]. Furthermore, the detection and data acquisition processes frequently require advanced CL analyzers or photodiodes integrated with signal converters, further driving up the cost and complicating the system design. These limitations restrict the practicality of microfluidic CL systems for applications requiring economical, compact, and portable glucose-monitoring devices. While they represent a step forward in glucose-sensing technology, their current implementation falls short of the flexibility and affordability needed for broader adoption [38].

To overcome these challenges, there is a pressing need for innovative approaches and strategies. The required advancements should focus on simplifying the fabrication processes, reducing the reliance on expensive materials and peripherals, and optimizing the device designs for cost-effective, portable, and user-friendly applications. By addressing these barriers, next-generation CL systems can better meet the demands of PoC and on-site testing, paving the way for more accessible and efficient glucose-monitoring solutions. Recently, machine learning (ML) and deep learning (DL) have emerged as transformative tools in addressing these limitations [39,40,41]. Deep learning as a subset of ML, has further advanced the field by eliminating the need for manual feature engineering. State-of-the-art research highlights the various computational models that enhance the accuracy, scalability, and efficiency of ECL biosensors by processing complex signal patterns and automating interpretation [42,43,44,45,46]. The emerging transformer-based architectures show potential in high-dimensional ECL signal analysis, particularly in scenarios involving complex multiplexing. These models excel at modelling long-range dependencies and promise significant improvements in signal classification accuracy. The integration of ML and DL into CL biosensors represents a paradigm shift, addressing the long-standing issues of signal interpretation, noise reduction, and multiplexing. By leveraging state-of-the-art models, other researchers have achieved advancements in sensitivity and portability, paving the way for next-generation biosensing platforms. The proposed deep learning-enhanced, portable chemiluminescence biosensor exemplifies this progress, harnessing DL models for real-time, high-precision analyte detection. Research efforts have successfully demonstrated the potential of integrating DL in portable biosensors to enhance accessibility, usability, and diagnostic capabilities. This work not only optimizes the analytical capabilities of CL, but also ensures scalability and affordability for widespread use in healthcare, environmental monitoring, and beyond.

In this study, paper-based WPµ-pad CL devices were successfully fabricated and validated for glucose sensing, highlighting the advantages of AI-based CL systems. A wax printing method was employed to create hydrophobic and hydrophilic zones on the paper-based substrate, forming a wax boundary to enhance the device structure. The analytical capabilities of the device were assessed using luminol/H_2_O_2_-based electrochemistry combined with glucose oxidase (GOx), a specific enzyme used for glucose detection. The underlying chemical reactions essential for glucose detection are elaborated through key equations referenced from prior studies. The results demonstrate that this 3DP-CL platform, when integrated with a smartphone, shows great promise for clinical diagnostics. It provides a highly sensitive and cost-effective approach for detecting a wide range of biomarkers. This integration of advanced electrochemical techniques with low-cost fabrication and smartphone connectivity presents a versatile solution for point-of-care testing (PoCT), potentially transforming diagnostic practices by enabling rapid, accessible, and reliable biomarker detection.

## 2. Materials and Method

### 2.1. Chemical Materials Used

Luminol (C_8_H_7_N_3_O_2_) ≥ 97% (HPLC grade), cobalt (II) chloride (CoCL_2_), sodium carbonate (Na_2_CO_3_), sodium bicarbonate (NaHCO_3_) ≥ 99.7%, hydrogen peroxide (H_2_O_2_), glucose oxidase (GOx), D-glucose, Lithium Lactate, cholesterol, creatinine, ascorbic acid, deionized water (DI), Whatman grade-1 paper, A KYLIE Pro Wax100 warmer hot wax heater, a VMS professional LM deluxe heavy-duty lamination machine, and a hot and cold A3 laminator were used.

### 2.2. CL Sensor Functionalization and Imaging Mechanism

**Figure 1:**
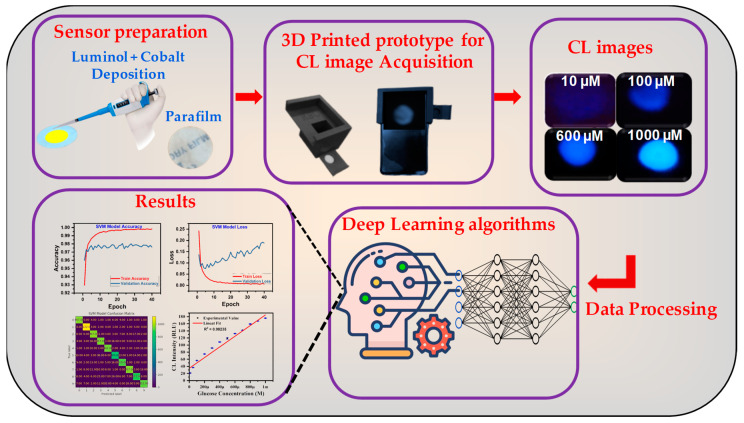
Process flow diagram of deep learning-enhanced portable chemiluminescence biosensor; illustration of key steps from bio-analyte detection to data analysis using 3D-printed, smartphone-integrated platform.

**Figure 2:**
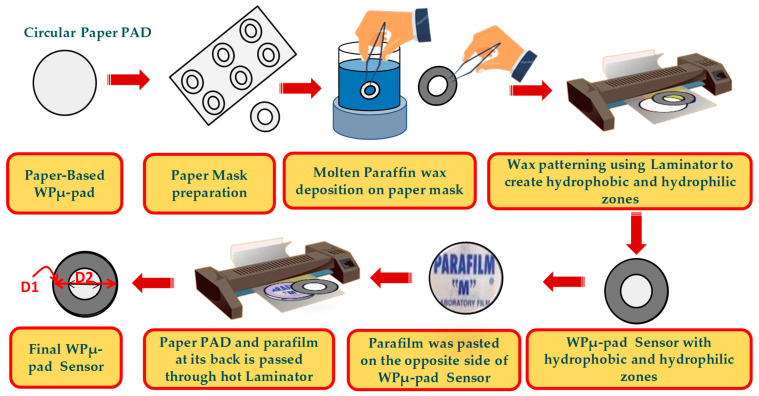
Step-by-step fabrication process of paper-based CL biosensor (D1 = 17 mm, D2 = 15 mm).

The assay procedure for the paper-based WPµ-pad CL biosensor is schematically illustrated in Figure 1 and detailed as follows: The prepared paper-based WPµ-pad CL biosensor was initialized by applying an optimized concentration of luminol (3 mM), followed by cobalt chloride (3 mM). The functionalized device was then incubated at 70 °C for 20–25 min to ensure proper activation. For glucose sample detection, varying concentrations of glucose (5 µL) were prepared and mixed in equal proportions with an optimized concentration of glucose oxidase (5 µL) before being added onto the sensing zone of the paper-based WPµ-pad device. The fabricated sensor was placed inside a 3D-printed black box for measurement and analysis, as depicted in Figure 2. Thanks to hydrophobic wax barriers, the solutions remained confined within a circular sensing area without spreading, allowing them to mix and react effectively. This ensured the enzymatic oxidation of glucose to hydrogen peroxide (H_2_O_2_), which subsequently triggered the chemiluminescent reaction.

The luminol/cobalt/GOx/glucose-based CL reaction is a modified system that uses glucose oxidase (GOx) and glucose to generate reactive oxygen species (ROS) for the luminol chemiluminescent reaction, without requiring external hydrogen peroxide (H_2_O_2_). This system is highly specific for glucose detection, making it valuable in biosensing and diagnostic applications. Glucose oxidase facilitates the oxidation of glucose by molecular oxygen, resulting in the formation of gluconic acid and hydrogen peroxide (H_2_O_2_) as a by-product [47,48], as illustrated in Equation (1):

(1) $$\mathrm{Glucose}+\mathrm{Oxygen}\;\overset{GOx}{\to }\;\mathrm{Gluconic}\mathrm{Acid}\;+\;\mathrm{Hydrogen}\mathrm{Peroxide}$$

Cobalt ions (Co^2^⁺) promote the breakdown of H_2_O_2_, leading to the production of reactive oxygen species, such as hydroxyl radicals (·OH) and singlet oxygen (^1^O_2_), as illustrated in Equation (2):

(2) $$\mathrm{Hydrogen}\;\mathrm{Peroxide}\;\overset{Cobalt\;ions\;({Co}^{2+})}{\to }ROS$$

ROS oxidize luminol, converting it to an excited-state intermediate (3-aminophthalate), as illustrated in Equation (3):

Luminol dianion L^2−^ + ROS ---------------> Excited 3-aminophthalate (3)

Excited 3-aminophthalate returns to its ground state, releasing a photon of light (~425 nm, blue glow), as illustrated in Equation (4):

Excited 3-aminophthalate ------------------> 3-aminophthalate + Photon (Light)(4)

A user-friendly and practical CL system suitable for real-world applications and operation by untrained users has been designed. This system features a 3D-printed black box that seamlessly integrates with smartphone devices, ensuring accessibility and ease of use. This innovative setup, depicted in Figure 1, demonstrates significant advancements in simplicity and practicality for non-expert users. The 3D-printed black box was meticulously designed to create a controlled and uniform environment for capturing CL signals. Constructed from black PLA material, the box effectively minimizes ambient light interference, ensuring consistent imaging conditions. Its interior is equipped with a holder for the reaction cuvette and an adjustable smartphone mount, allowing for the precise alignment of a smartphone camera with the reaction chamber. The straightforward design ensures ease of replication and operation, requiring no technical expertise. This prototype allows users to capture real-time images of the CL signals directly using their smartphone cameras. The captured images are then processed and analyzed using advanced deep learning models designed to accurately predict glucose concentrations. For these experiments, we employed commonly available smartphones, including Android (e.g., Samsung Galaxy series) and iOS (e.g., iPhone models) types, showcasing the system’s compatibility across a wide range of devices. The smartphone was securely placed in the designated mount within the black box, ensuring optimal alignment with the reaction chamber. CL signal images were captured using the default camera application with pre-configured settings, such as fixed exposure, ISO, and focus, standardized during preliminary testing to deliver consistent results.

Although minor variations in camera specifications, such as resolution and sensitivity, may affect raw image quality across different smartphone models, these differences are effectively addressed during the pre-processing stage of the deep learning pipeline. The algorithm normalizes the input images to ensure reliable and accurate glucose concentration predictions, regardless of device-specific characteristics or slight variations in the image acquisition conditions.

This streamlined approach not only enhances the efficiency of glucose detection, but also simplifies data interpretation, making it accessible to individuals without specialized training. By combining the portability of smartphone technology with the precision of deep learning algorithms, this system demonstrates significant potential for real-world applications in healthcare and diagnostics, bridging the gap between laboratory innovations and everyday usability.

### 2.3. Fabrication of Paper-Based CL Sensor

Whatman filter paper (Grade-1) was utilized to fabricate a paper-based WPµ-pad CL biosensor for glucose detection. The wax printing method was applied to define distinct hydrophilic and hydrophobic zones on the paper substrate. This approach is characterized by its simplicity, cost-effectiveness, and adaptability, making it highly suitable for translating biosensor technology from controlled laboratory environments to practical, real-world applications. The fabrication process was carried out methodically, as detailed below. First, a laser cutter was used to prepare a mask, which was then aligned precisely over the circular shaped paper substrate having a diameter (D1) of 17 mm. A wax barrier was created by cutting A4 paper into the desired shape using a laser cutter and immersing it in molten wax. The wax-coated paper was then precisely placed over the specified conductive areas, forming an effective outer boundary around the inner circular pad. This process ensured the accurate alignment and functionality of the barrier. Subsequently, the assembly was passed through a hot laminator to ensure the proper integration of the hydrophobic and hydrophilic zones.

To enhance the structural stability of the biosensor, parafilm coating was applied as the final step. This provided additional durability to the device, making it robust for testing and application. The step-by-step fabrication process is visually represented in Figure 2. By leveraging these wax-printing methods, the CL biosensor was successfully fabricated and demonstrated effective glucose detection capabilities, showcasing its potential for practical use in biosensing applications. This process exemplifies the utility of combining simple, yet efficient techniques to advance the development of paper-based diagnostic tools.

## 3. Results and Discussion

### 3.1. Optimization for CL Biosensor

**Figure 3:**
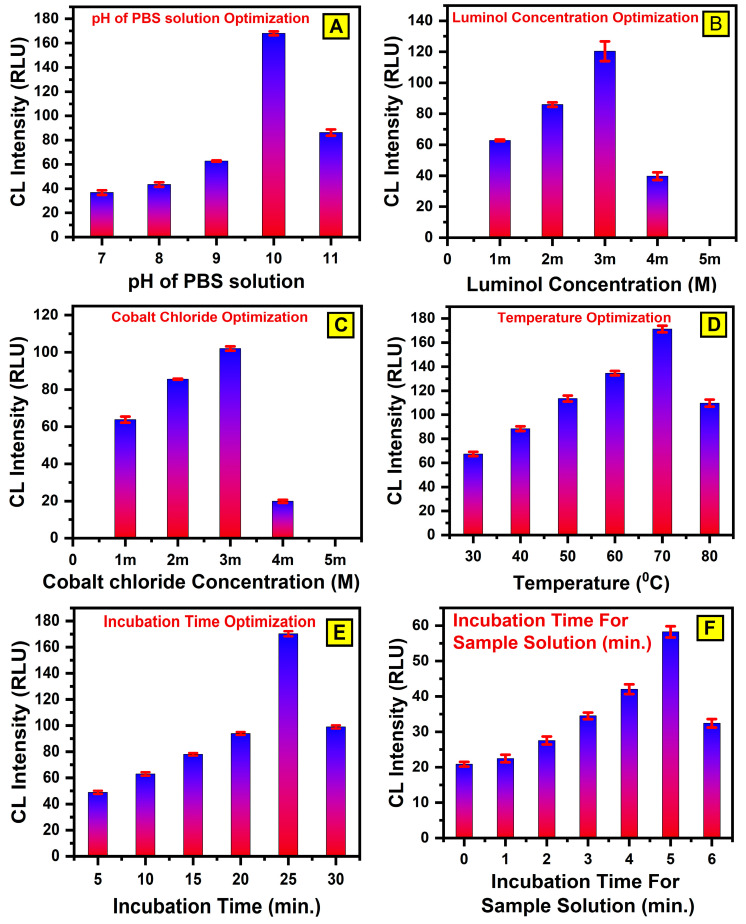
(**A**) pH value optimization of PBS solution, which was used as substrate solution (luminol/cobalt (II) chloride); optimum value 10 pH for PBS solution was used. (**B**) Luminol concentration optimization (optimum value of 3 mM). (**C**) Cobalt concentration optimization (optimum value of 3 mM). (**D**) Temperature optimization: incubation temperature varied from 30 °C to 80 °C for luminol (3 mM), H_2_O_2_ (1 mM), and cobalt chloride (3 mM). (**E**) Optimized incubation time of 25 min for drying deposited luminol (3 mM) and cobalt chloride (3 mM) at temperature of 70 °C was determined. (**F**) Incubation time optimization for sample solution (glucose/GOx) before being applied to sensor. Error bars indicate that all experiments were conducted independently on three different devices (n = 3).

The optimization of luminol, cobalt ions, pH, and temperature is crucial for enhancing the sensitivity and reliability of CL detection. Fine-tuning these parameters ensures precise analyte analysis and quantification, making the method adaptable for a wide range of applications. The CL reaction on the paper-based WPµ-pad surface is significantly influenced by these parameters. Luminol/cobalt/H_2_O_2_-based CL reactions are known to perform best under alkaline conditions [49]. Therefore, investigating the pH effect of the CL reagents is crucial. In this study, glucose and enzyme solutions were formulated in phosphate-buffered saline (PBS) at a pH of seven, while the luminol-based substrate solution was prepared in PBS adjusted to a pH of ten. To assess the influence of pH on CL intensity, the experiments were conducted with substrate solutions at pH levels of eight, nine, and ten. As illustrated in Figure 3A, the highest CL intensity was observed at a pH of 10 with three devices (n = 3), confirming its suitability for maximizing the reaction signal. Based on these findings, the subsequent experiments utilized PBS at pH ten to prepare substrate solutions, and to prepare sample solutions, we used PBS at a pH of seven. This careful optimization of pH ensures an enhanced CL performance, paving the way for reliable and precise detection in biosensing applications.

The concentrations of luminol and cobalt chloride, as the key luminescent reagents, significantly influence the CL response. To optimize the CL signal, the impact of luminol concentration was examined across a range from 1 mM to 4 mM, as depicted in Figure 3B for three devices (n = 3). The findings revealed a gradual increase in the CL signal as the luminol concentration rose from 1 mM to 3 mM, indicating improved luminescence efficiency within this range. However, a decline in the CL signal was observed at concentrations exceeding 3 mM. This decrease is attributed to the self-quenching effect, a phenomenon where higher luminol concentrations lead to reduced light emission due to molecular interactions that inhibit the luminescence process, consistent with the previous reports [50]. Based on this analysis, the optimal luminol concentration for the subsequent experiments was determined to be 3 mM. Similarly, the effect of cobalt chloride concentration on the intensity of CL signal was evaluated, while maintaining a constant luminol concentration. The concentration of cobalt chloride was adjusted within the range from 1 mM to 4 mM, as shown in Figure 3C, to evaluate its effect on CL intensity. The results demonstrated a steady increase in CL intensity as the cobalt concentration increased from 1 mM to 3 mM. This trend highlights cobalt chloride’s catalytic role in enhancing the reaction, likely by promoting the decomposition of hydrogen peroxide and generating reactive oxygen species that drive the luminescence process. However, beyond 3 mM, any further changes in concentration showed minimal impact, suggesting an optimal concentration range for maximizing the CL signal. Beyond 3 mM, a gradual decline in CL intensity was observed, possibly due to similar quenching effects or the saturation of catalytic sites. Therefore, the optimal concentrations for both the reagents were identified as 3 mM with three devices (n = 3). These optimized conditions ensure a robust and sensitive CL response, laying a solid foundation for accurate and reliable detection in various biosensing applications.

The impact of temperature and incubation time on the luminol- and cobalt-functionalized, paper-based sensor pads were systematically investigated, as both these parameters significantly influence CL intensity. To analyze the impact of drying temperature, the functionalized, paper-based WPµ-pads were dried at temperatures ranging from 30 °C to 80 °C for a fixed time interval of 10 min with the three devices (n = 3). As illustrated in Figure 3D, the results showed that the CL intensity increased progressively as the drying temperature rose, with the maximum intensity observed at 70 °C. Beyond 70 °C, the CL intensity began to decrease, likely due to the degradation of sensitive reagents or structural changes in the substrate. Based on these findings, 70 °C was considered as the optimal drying temperature for the subsequent experiments. The impact of incubation time was also studied at the optimal drying temperature of 70 °C. Six different incubation durations ranging from approximately 5 min to 30 min were tested. After the luminol and cobalt (II) chloride solutions were applied to the detection zone, an assay was performed to measure the CL intensity. The results indicated that the CL intensity increased steadily with longer incubation times, reaching a peak at 25 min. Beyond 25 min, the intensity began to decline, suggesting that prolonged incubation could lead to reagent evaporation or substrate degradation, which would negatively impact the CL reaction. Based on these findings, an incubation time of 25 min at a drying temperature of 70 °C was determined to provide an optimal analytical performance, as illustrated in Figure 3E for the three devices (n = 3). This combination yielded the highest CL intensity, ensuring maximum sensitivity and reliability for detecting H_2_O_2_ in the reaction zone.

Apart from reagent concentration and buffer pH, the incubation time between glucose and glucose oxidase (GOx) before being applied to the sensor significantly impacts the CL intensity of the proposed system. To assess this, seven incubation durations ranging from approximately 0 to 7 min were examined with the three devices (n = 3). For the shortest incubation time (~0 min), an assay was performed immediately after the solutions were mixed and applied to the detection zone. As illustrated in Figure 3F, the results showed a steady increase in chemiluminescent intensity as the incubation time increased, reaching a peak at 5 min. This suggests that the enzymatic reaction between glucose and GOx, leading to the formation of gluconic acid and hydrogen peroxide (H_2_O_2_), was most effective within this period. Beyond 5 min, the CL intensity stabilized, indicating that the enzymatic oxidation process had reached its maximum efficiency. Based on these findings, an incubation time of 5 min was selected as the optimal duration. This timeframe balances achieving maximum CL intensity, and the assay process is rapid, ensuring the method is both efficient and practical for real-world applications. The 5 min incubation time provides a reliable analytical performance, while supporting the sensor’s rapid and effective detection capabilities.

### 3.2. Analytical Performance of Paper-Based CL Biosensors

**Figure 4:**
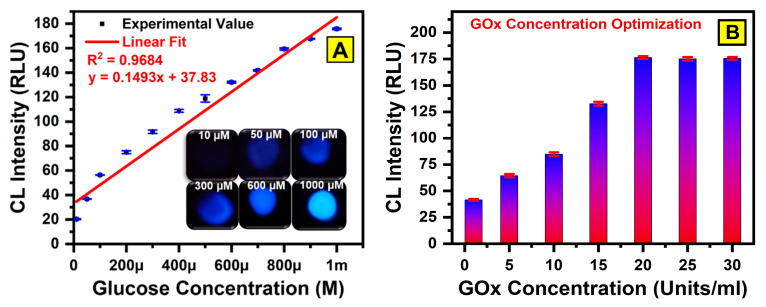
(**A**) Detection of glucose using WPµ-pad paper-based (CL) device. (**B**) Optimization of glucose oxidase (GOx) activity by varying GOx concentration (1–30 Units/mL), with fixed parameters for glucose concentration (1 mM), cobalt chloride (3 mM), and luminol concentration (3 mM).

The analytical performance of the developed paper-based CL sensor for glucose detection was assessed under optimized conditions. Figure 4A illustrates the correlation between CL intensity and various glucose concentrations. The results show a consistent increase in CL intensity as the glucose concentration rises, confirming the sensor’s ability to provide quantitative glucose measurements within the linear range from 10 µM to 1000 µM, with a correlation coefficient (R^2^) of 0.9684. This demonstrates the sensor’s capability for accurate quantitative analysis over a wide concentration range. The limit of detection (LOD) was determined to be 8.68 µM, defined as the lowest glucose concentration that generated a signal three standard deviations above the background noise. This low detection limit reflects the high sensitivity of the sensor. The ability to detect glucose accurately at such low concentrations underscores the sensor’s precision, making it suitable for applications requiring reliable glucose detection. These findings validate the paper-based WPµ-pad CL sensor as an effective tool for glucose monitoring, offering both high sensitivity and precision. The clear linear relationship between CL intensity and glucose concentration, along with the calculated LOD, further highlight the sensor’s potential for real-time, quantitative glucose measurement in various settings.

The sensor relies on a luminol/cobalt/H_2_O_2_-based chemical reaction, showcasing its versatility in glucose-monitoring applications. To optimize the glucose oxidase (GOx) concentration, experiments were conducted by varying the GOx levels from 1 Unit/mL to 30 Units/mL. The results showed a gradual increase in CL intensity, which reached saturation at 20 Units/mL. Therefore, a concentration of 20 Units/mL was selected as the optimal level for all the subsequent experiments. Figure 4A provides a comprehensive overview of the calibration curve for glucose concentration, and an optimization curve for GOx concentration is illustrated in Figure 4B. These results validate the performance of the proposed CL sensor and its ability to deliver accurate and reliable results for glucose detection.

### 3.3. Repeatability, Stability, and Interference Study with CL Biosensor

**Figure 5:**
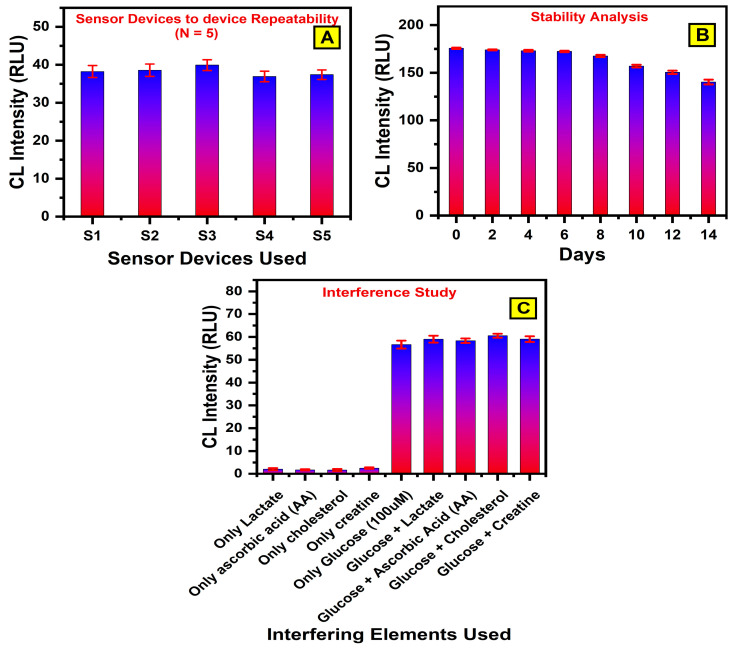
(**A**) Analysis of device-to-device repeatability conducted using glucose (50 µM), glucose oxidase (GOx, 20 Units/mL), luminol (3 mM), and cobalt chloride (3 mM). (**B**) Stability evaluation performed with glucose (1 mM), GOx (20 Units/mL), luminol (3 mM), and cobalt chloride (3 mM). (**C**) Interference study assessed using glucose (100 µM), alongside potential interferents, including lactate, ascorbic acid, cholesterol, and creatine (500 µM each). Error bars represent data from three independent experiments conducted separately.

In CL studies, it is essential to ensure that the fabricated device exhibits reproducibility, stability, and robustness to obtain reliable and consistent results. Reproducibility refers to the consistency of the outcomes when an experiment is repeated multiple times, either using the same device or different devices. To evaluate the reproducibility of the developed CL sensor, five independent devices sourced from three distinct production batches were tested under identical conditions. As shown in Figure 5A, these devices were subjected to the same experimental procedures to assess their performance and ensure that the results could be reliably replicated across different samples. By testing multiple devices from different production batches, this study aimed to determine if there were any variations in device performance due to batch-to-batch differences or fabrication inconsistencies. The reproducibility test involved conducting chemiluminescence measurements on all five devices under the same conditions to assess their ability to produce consistent CL signals. The outcomes of these tests were analyzed to verify whether each device generated similar results, highlighting the stability and reliability of the sensor. The luminescent responses of these devices based on luminol (3 mM)/cobalt chloride (3 mM)/Gox (20 Units/mL)/glucose (50 µM) were analyzed. The relative standard deviations (RSDs) across these devices remained below 5% (4.13%, 4.23%, 3.61%, 3.57%, and 3.39%), indicating excellent device-to-device repeatability.

Stability studies were conducted to assess the long-term performance and shelf life of the CL biosensor, ensuring its functionality remains reliable over time. For this evaluation, three biosensor devices were fabricated and primed with luminol (3 mM)/cobalt (II) chloride (3 mM) in dry form at 70 °C. The devices were then tested periodically over 14 days, with the chemiluminescent signal recorded after each use, as shown in Figure 5B. To minimize the risk of contamination, the devices were securely sealed and stored at room temperature between the experiments. The results demonstrated that the biosensor maintained over 98% of its initial chemiluminescent signal during the first six days, indicating excellent short-term stability. However, a decline in performance became evident after the 14th day, as the signal intensity reduced significantly to approximately 15% of its original value by the end of the testing period. These findings suggest that the biosensor exhibited exceptional stability and reliability during the initial two weeks, but its performance diminished noticeably beyond this timeframe. This study highlights the biosensor’s capacity to provide consistent and reproducible results for at least two weeks under appropriate storage conditions. This level of stability makes it highly suitable for practical applications, particularly in analytical and diagnostic contexts where reliability and reproducibility are crucial. The observed stability reinforces the device’s potential as a robust and dependable tool for use in various scenarios, ensuring a high-level performance during its functional lifespan.

To assess the anti-interference capabilities of the CL device, selectivity analysis was conducted. This is an essential step in identifying and addressing the potential factors that may interfere with the accuracy and specificity of CL biosensors. Common substances that could potentially act as interferents, such as lactate, ascorbic acid, cholesterol, and creatine, were selected for testing. These compounds are commonly present in human biological fluid, such as blood, sweat, and saliva, where bio-analytes like glucose, urea, or creatinine are typically measured. Their presence at similar concentrations can interfere with detection processes, making them critical for evaluating biosensor specificity. The inclusion of these substances is standard practice in the validation of biosensors to ensure accuracy and reliability. Potential interference from these compounds could lead to false positives or negatives in real-world applications.

By testing the biosensor against these commonly encountered interferents, we can robustly evaluate its ability to differentiate the target analyte (glucose) from other coexisting species. This ensures the biosensor’s specificity and enhances its practical utility in clinical and diagnostic scenarios. The experiment began by measuring the CL signal in the samples that did not contain glucose. As expected, no chemiluminescence was detected in these samples, indicating that the sensor did not produce any signal in the absence of glucose. This outcome is consistent with the behavior of the luminol/cobalt system combined with glucose oxidase (GOx), which only generates light when glucose is present. The lack of signal in non-glucose samples serves to confirm the high specificity of the reaction, ensuring that the sensor will only respond to the target analyte (glucose) and not to the other common substances. These results demonstrate the device’s robustness and selective response, ensuring that it can accurately measure glucose levels without interference from the other substances commonly found in the biological samples.

Subsequently, CL intensity was measured for 100 µM glucose under optimized conditions, as outlined in Section 3.2. Separate tests were then conducted to evaluate interference by pairing glucose with each individual compound, glucose and urea, glucose and ascorbic acid, glucose and cholesterol, and glucose and creatine. For each test, CL images were captured, and the relative standard deviation (RSD) was calculated to assess the device’s consistency. The RSD values for all the potential interfering compounds were found to be below 5%, as shown in Figure 5C. These findings demonstrate that the CL device maintains excellent selectivity and is not significantly affected by the presence of common interfering substances, such as lactate, ascorbic acid, cholesterol, and creatine. This highlights the device’s reliability and its ability to deliver accurate results despite the presence of these common compounds. The findings demonstrate that the device maintains high specificity for glucose detection, even in the presence of potentially interfering analytes. This robustness makes the biosensor highly reliable for practical applications where the sample compositions may vary, ensuring accurate and interference-free measurements.

## 4. Deep Learning for CL Sensor Validation

### 4.1. Dataset Statistics

After evaluating the analytical performance, more than 600 experiments were conducted to develop and test deep learning-enhanced, machine learning models using CL images. The dataset statistics detailed in Table 1 provide valuable information about its structure and distribution, which are essential for effective model training. To overcome the challenge of a limited dataset, data augmentation techniques were applied to the image dataset before training the models. Data augmentation is crucial when training deep learning models with limited data, as it helps artificially expand the size and diversity of the training set. This technique involves transforming the original data in various ways to create new, varied examples for the model to learn from. For this study, the ImageDataGenerator utility from the Keras library was used to perform several types of transformation of the images, such as rotation, scaling, and flipping. These transformations introduce variations into the dataset, providing the model with exposure to a wider range of scenarios and conditions, which improves its generalization capability for new, unseen data.

By augmenting the dataset, the model benefits from a richer and more diverse set of training examples. This not only strengthens the model’s ability to adapt to various real-world situations, but also reduces the risk of overfitting. Overfitting occurs when the model becomes too tailored to the specific patterns in the training data, causing it to perform poorly on new, unseen data. Data augmentation helps prevent this issue by ensuring that the model learns broader, more generalizable features. Ultimately, the augmented dataset provides a more comprehensive representation of the target domain, improving the model’s adaptability and robustness. This leads to more accurate predictions in diverse conditions and enhances the model’s overall effectiveness and reliability across a wide range of real-world applications.

The model configuration includes several augmentation and training parameters to improve its performance and generalization. ImageDataGenerator is set up with hyperparameters that pre-process the input data effectively. One key transformation is rescaling the pixel values to a range from 0 to 1 (rescale = 1/255), which normalizes the data and enhances numerical stability during training. The input images are resized to 128 × 128 pixels (img_size), ensuring uniformity in dimensions regardless of the original image size. The batch size is configured to 32, meaning the model processes 32 samples per training iteration, balancing computational efficiency and gradient stability. This combination of pre-processing ensures that the model can handle diverse input data more effectively. Real-time augmentation dynamically applies transformations to the images during training, eliminating the need for additional storage for augmented datasets. This approach increases the diversity of the training set without manually expanding the dataset, enabling the model to generalize unseen data better. As a result, the network is exposed to variations that enhance its ability to handle noise and distortions in real-world scenarios. The training architecture further integrates several advanced parameters. The input shape is 128 × 128, with an Adam optimizer and a learning rate of 0.001. The pooling method is AveragePooling, with activation functions set to ReLU and softmax. Dropout is applied at a rate of 0.5 to prevent overfitting, while the padding method is ZeroPadding. Training spans 40 epochs, with metrics focused on accuracy and a loss function defined as categorical cross entropy. This combination of augmentation and architectural design ensures robust model training and performances.

### 4.2. Various Deep Learning Model’s Implementation

In the proposed work, we have tested the dataset with various deep learning models, including K-Nearest Neighbors (KNN), Random Forest, InceptionV3, VGG16, ResNet-50, and support vector machine (SVM). Among the DL methods, SVMs have been widely used for classification tasks, such as distinguishing analytes in noisy environments. SVMs perform well with small datasets, offering high specificity.

SVMs are powerful and versatile machine learning models designed for both classification and regression tasks. The primary mechanism of SVM involves finding an optimal decision boundary, known as a hyperplane, that separates data points belonging to different classes. The process begins with the input of labeled data points, each characterized by specific features. For datasets that are not linearly separable, SVM utilizes kernel functions to transform the data into a higher-dimensional space where a linear hyperplane can effectively separate the classes. The model then identifies the optimal hyperplane by maximizing the margin, which is the distance between the hyperplane and the closest data points from each class, referred to as support vectors. This margin maximization ensures the hyperplane is as far as possible from the nearest data points, improving the model’s generalization. SVM also excels at handling non-linear data by leveraging various kernel functions, such as linear, polynomial, and radial basis functions, to implicitly map the data into a higher-dimensional space. During prediction, SVM assigns a new data point to a class based on its position relative to the hyperplane in classification tasks or predicts a numerical value for regression. This systematic approach makes SVM highly effective for high-dimensional and complex datasets across various applications [51,52].

Random Forests and ensemble methods like gradient boosting have also been applied to improve the signal-to-noise ratios in ECL systems. They function by creating an ensemble of decision trees, combining their outputs to enhance accuracy and reduce the risk of overfitting. The process begins with dataset sampling, where the training data are randomly sampled with replacement through a technique called bagging (bootstrap aggregation). This creates multiple subsets, each used to train an individual decision tree. To further introduce diversity, a random subset of features is selected at each node to determine splits. Each decision tree is grown to its maximum depth or until a stopping criterion is met, without pruning, allowing for the trees to fully capture patterns within their respective subsets. During prediction, the Random Forest aggregates results from all the trees; for classification, it uses a majority-vote system to decide the final class, and for regression, it calculates the average of the tree outputs. This ensemble approach effectively reduces variance by averaging predictions across multiple trees, making the model more robust and less sensitive to noise in the data. Additionally, the randomness in feature and data subset selection addresses bias, improving generalization. Random Forests are highly effective for large, high-dimensional datasets and are widely applied in fields like medical diagnostics and image recognition. These methods are robust to overfitting and excel in tasks requiring analyte quantification [53,54,55].

Another notable DL method, KNN, has been used in simpler CL applications for identifying analytes based on historical data, but struggles with computational efficiency in large-scale or real-time applications. The process begins by selecting the number of neighbors (k) and calculating the distance between the test and training data points using either the Manhattan or Euclidean distance. The nearest data points are identified by sorting these distances in ascending order. For regression tasks, the model predicts the value for the test data point by calculating the mean of the k-nearest neighbors. For classification, the test data point is assigned the class label based on the majority class among its k-nearest neighbors. The class with the highest frequency in the neighborhood effectively “votes” to determine the classification outcome [56,57].

InceptionV3 is a sophisticated convolutional neural network (CNN) architecture known for its ability to extract meaningful patterns from complex datasets, particularly in high-resolution imaging tasks. The mechanism of InceptionV3 is built upon several key innovations that enhance its performance and computational efficiency, making it highly effective for analyzing spatial patterns, such as those found in CL imaging. The core of InceptionV3 lies in its Inception modules, which process input data through multiple convolutional layers of varying kernel size (e.g., 1 × 1, 3 × 3, and 5 × 5) in parallel. This design enables the network to capture features at different scales, such as fine details and broader structures, within the same layer. By integrating the information from multiple receptive fields, the model gains a comprehensive understanding of spatial hierarchies in the input data. To reduce the computational costs, 1 × 1 convolutions are employed for dimensionality reduction, effectively compressing the feature maps before applying more computationally expensive operations. To ensure stability during training, InceptionV3 extensively uses batch normalization, which normalizes the layer outputs, accelerates convergence, and mitigates issues like vanishing or exploding gradients. In the context of CL imaging, InceptionV3’s deep architecture and multi-scale feature extraction make it highly effective at identifying fine-grained luminescent patterns, quantifying bio-analytes with precision. Its ability to model complex relationships, while remaining computationally efficient ensures its suitability for high-resolution datasets and real-world applications [58,59].

VGG16 is a deep convolutional neural network architecture known for its simplicity and effectiveness in image recognition tasks. Its mechanism is based on sequential layers of small 3 × 3 convolutional kernels, which capture spatial features, while maintaining computational efficiency. Each convolutional layer is followed by ReLU activation to introduce non-linearity, and the max-pooling layers reduce spatial dimensions, retaining essential features. The network consists of 16 weighted layers, including fully connected layers at the end for classification. VGG16’s consistent architecture, with uniform kernel sizes and layer depths, enables it to model complex patterns in data, making it suitable for high-resolution image analysis. Its simplicity and uniform architecture make it an excellent choice for analyzing structured luminescent patterns. However, its high computational cost can be a drawback for real-time applications or systems with limited computational resources [42,60,61].

ResNet-50 is a deep convolutional neural network architecture that introduced the concept of residual learning to overcome the challenges of training very deep networks, such as vanishing gradients and a degraded performance. The architecture consists of 50 layers, organized into a series of convolutional and pooling layers grouped into residual blocks. Each residual block includes a set of convolutional layers, followed by batch normalization and ReLU activation. What makes ResNet-50 unique is its use of shortcut connections, which bypass one or more layers and directly add the input of a block to its output. This addition forms a residual mapping that allows for the network to learn the difference between the input and the desired output, rather than full transformation. By preserving critical information across layers, these connections ensure efficient gradient flow during backpropagation, even in very deep networks. The architecture employs bottleneck layers, which use 1 × 1 convolutions to reduce, and then restore dimensionality within blocks, minimizing the computational costs, while maintaining representational power. ResNet-50 also includes global average pooling and fully connected layers at the end for classification tasks. Its ability to train extremely deep networks with high accuracy makes it ideal for tasks like image recognition and object detection in large, complex datasets [62,63].

In this work, the dataset was divided into two subsets with an 80:20 split for training and testing, ensuring an effective evaluation of the model’s performance. The dataset included 10 unique classes, representing diverse visual categories. The training set comprising 80% of the data allowed for the model to learn meaningful patterns and features within the images. Through iterative adjustments of its parameters during training, the model refined its ability to make accurate predictions. The remaining 20% of the data, designated as the testing set, served to evaluate the model’s generalization capability. This step ensured that the model could accurately classify new, unseen data, reflecting its real-world applicability. By exposing the model to a variety of examples within each of the 10 classes, it developed the ability to distinguish between distinct visual features, enabling robust categorization. The inclusion of 10 distinct classes introduced diversity to the dataset, challenging the model to recognize a wide range of image patterns and characteristics. This multi-class setup encouraged the model to learn complex decision boundaries, which are critical for handling real-world classification tasks involving multiple categories. Overall, the 80:20 split strategy balanced the need for effective training and comprehensive testing. The training set provided sufficient data to fine-tune the model’s weights and biases, while the testing set offered an unbiased assessment of its performance on unseen examples. This structured approach ensured that the model not only learned to classify the training data accurately, but also developed the versatility to perform well across a variety of new and diverse inputs, thereby enhancing its overall reliability and effectiveness.

### 4.3. Comparison of Various Standardized Deep Learning Models Used

**Figure 6:**
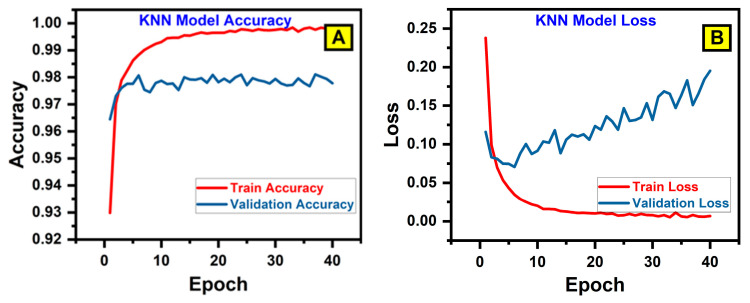

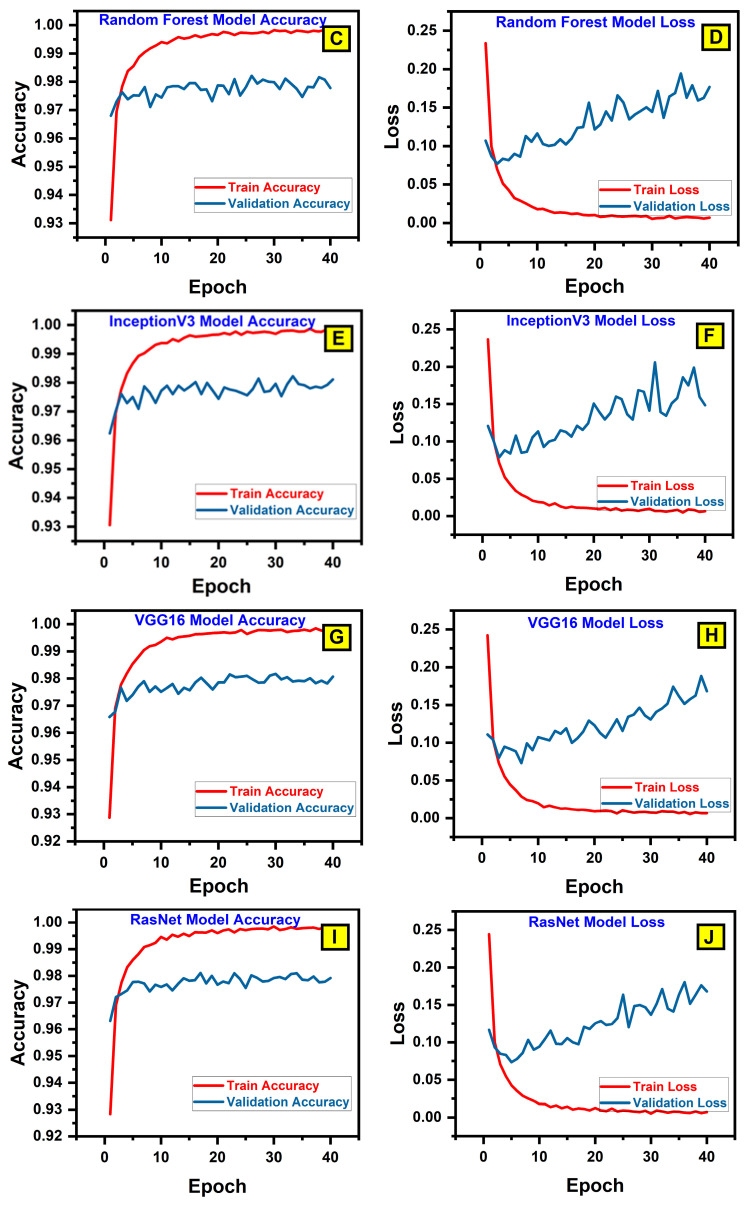

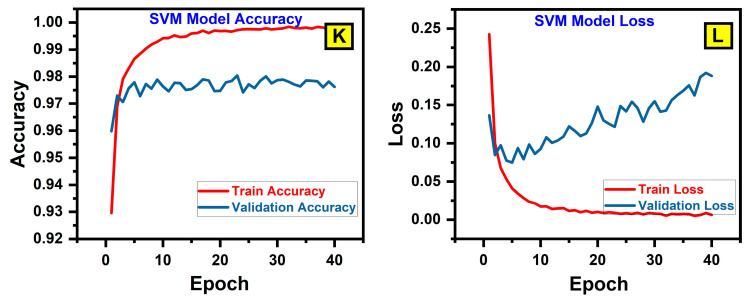
A graphical comparison of the different benchmark models based on their accuracy and loss metrics for (**A**) KNN model accuracy, (**B**) KNN model loss, (**C**) Random forest model Accuracy, (**D**) Random forest model loss, (**E**) Inception V3 model accuracy, (**F**) Inception V3 model loss, (**G**) VGG16 model accuracy, (**H**) VGG16 model loss, (**I**) RasNet model accuracy, (**J**) RasNet model loss, (**K**) SVM model accuracy, (**L**) SVM model loss.

Table 2 illustrates the comparative evaluation of various deep learning models for predicting glucose concentration showcases distinct performance patterns, highlighting their strengths and limitations. The models analyzed include KNN, Random Forest, InceptionV3, VGG16, RasNet-50, and SVM, as shown in their respective accuracy and loss curves as illustrated in Figure 6 (A-L respectively). The SVM model consistently outperforms the others, achieving a peak validation accuracy of 99%. This high accuracy is attributed to its margin-based optimization approach, which effectively separates classes and handles complex data distributions. The stability of SVM is further evidenced by minimal fluctuations in validation loss, reflecting robust generalization to unseen data and its capacity to resist overfitting. These characteristics make SVM ideal for predictive analysis requiring high precision.

The InceptionV3 model, based on deep transfer learning, also delivers an impressive performance, with a validation accuracy nearing 99%. InceptionV3 employs depth-wise separable convolutions, significantly reducing the trainable parameters, while maintaining feature extraction efficiency. This design enables it to generalize well, as observed in its nearly parallel training and validation loss curves. Its computational efficiency and robust architecture make InceptionV3 a reliable alternative for scenarios requiring a balance between accuracy and computational resources. RasNet-50 demonstrates a strong predictive ability, achieving a validation accuracy of approximately 98.8%. Its modular and scalable architecture adapts well to data variations, which contributes to its consistent performance. The generalization capability of RasNet is evident from the relatively narrow gap between training and validation losses, making it a viable option in applications demanding adaptability.

The Random Forest model performs moderately well, achieving a validation accuracy of around 98.2%. Its ensemble-based decision tree approach ensures a stable performance, but its effectiveness is slightly constrained by increasing validation loss, suggesting susceptibility to overfitting under specific conditions. However, its robustness in handling noisy data makes it a reliable choice for simpler datasets. KNN (K-Nearest Neighbors) exhibits a reasonable performance, with a validation accuracy of ~98%. However, its sensitivity to data distribution and lack of feature learning contribute to noticeable overfitting, as seen from the widening gap between the training and validation loss curves. While simple and intuitive, KNN is less suitable for datasets with complex patterns or large feature spaces. The VGG16 model, a deeper convolutional neural network, achieves a validation accuracy of around 98%. However, its complex architecture introduces challenges in generalization, leading to significant overfitting. The increasing validation loss curve reflects this limitation, as the model struggles to adapt to unseen data. Despite its strong feature extraction capability, its inefficiency in balancing training and validation losses makes it less practical for large-scale applications. SVM stands out as the most reliable model due to its exceptional accuracy and stability, making it the preferred choice for glucose concentration prediction. InceptionV3, with its computational efficiency and high accuracy, is a strong alternative, especially for resource-constrained environments. RasNet also performs robustly, particularly in applications requiring scalability. While KNN, Random Forest, and VGG16 provide acceptable results, their limitations—such as overfitting and lack of generalization—make them less suited for demanding predictive tasks. These insights emphasize the critical role of model selection in optimizing predictive accuracy and reliability for real-world applications.

## 5. Analysis and Validation of an Unknown Glucose Sample Using Support Vector Machines

To assess the practical performance of the developed device, we tested its capability to predict glucose concentrations in unknown samples using deep learning methods, as illustrated in Table 3. Five glucose solutions with concentrations falling within the linear detection range were prepared using commercially available D-glucose. These samples were initially analyzed in the laboratory to determine their precise concentration values, which served as the reference data for comparison. The reference data were then used to evaluate the accuracy and reliability of the device’s predictions. This testing ensured that the device could effectively quantify glucose concentrations in real-world scenarios. Next, we tested the same set of samples using the fabricated WPµ-pad CL device. For each glucose concentration, CL images were captured. The concentration of the unknown glucose samples was calculated using a calibration curve generated by the device. The values obtained from the WPµ-pad CL device were found to align closely with the results derived from laboratory analysis, thereby validating the reliability of the device’s measurements.

To further enhance the glucose concentration prediction process, we employed a deep-learning model, specifically SVM, to analyze the captured CL images. The CL images were fed into the SVM model, which predicted the glucose concentrations of the unknown samples. The predicted concentrations were then compared with both the laboratory reference values and the results obtained from the WPµ-pad CL device.

Mean Absolute Error (MAE) evaluates regression models by averaging absolute differences between predicted (Y_P_) and actual (Y_A_) values. It is robust, interpretable, and was calculated as follows:

(5) $$MAE=\frac{1}{n}\sum_{i=1}^{n}\left|Y_{Pi}-Y_{Ai}\right|\;\;\;$$

Remarkably, the predictions generated by the deep learning model closely matched the laboratory-measured values, as well as those from the WPµ-pad CL system, demonstrating the robustness of the combined approach. These findings underscore the potential of the WPµ-pad CL device when integrated with deep learning to deliver highly accurate glucose concentration predictions in real-time scenarios. By combining hardware efficiency with advanced data analysis techniques, this system provides a reliable solution for glucose monitoring. The detailed analysis of the unknown glucose samples and the corresponding results are presented in Table 3. This study highlights the practicality and effectiveness of integrating deep learning models like SVM into CL-based sensing devices, paving the way for their application in point-of-care testing and other real-world scenarios.

Table 4 highlights the application of CL-based sensing materials for detecting various analytes, focusing on their performance metrics, such as linear range, the limit of detection (LOD), and the integration of artificial intelligence (AI) algorithms. For glucose detection, the HCC/HLG film exhibits exceptional sensitivity with dual linear ranges (0.01–50 nM and 50 nM–2.0 μM) and an extremely low LOD of 9.0 pM. This sensitivity, however, was achieved without AI support. Similarly, microplates and CCGTSs target glucose with broader linear ranges of 0.1–2.5 mmol L−1 and 0.1–100 mM, respectively. Their LODs of 120 µmol L−1 and 0.0948 mM suggest utility in higher concentration monitoring, but again AI algorithms were not employed.

For cortisol detection, LFIA demonstrates a linear range of 0.3–60 ng/mL and a low LOD of 0.3 ng/mL, making it suitable for hormonal analysis. Similarly, Cu-MOXs are used for dopamine sensing, showcasing high sensitivity with a linear range of 40–200 nM and an LOD of 10 nM. Both systems, however, lack AI integration. MOFs for glucose detection stand out for their broad linear range (0.2–100 mmol L^−1^) and low LOD (0.011 mM), demonstrating efficacy in high-concentration glucose monitoring. However, these systems do not incorporate AI. In contrast, the WPµ-pad distinguishes itself by integrating AI, specifically deep learning, to enhance glucose detection. With a linear range of 10–1000 µM and an LOD of 8.68 µM, it balances sensitivity with practicality. The incorporation of AI improves its analytical capabilities, enabling real-time application and making it a standout among CL-based sensors. This comparison underscores the importance of integrating AI in sensor technologies to achieve enhanced accuracy, versatility, and adaptability for real-world applications.

## 6. Conclusions

This study presents the development of a cost-effective and portable CL platform enhanced with deep learning capabilities for efficient and accurate analyte detection. This system is designed around a smartphone-based platform, leveraging its accessibility and computational power for CL imaging. The innovative design uses wax printing technology to fabricate hydrophilic and hydrophobic zones on a paper-based substrate, enabling precise control over fluid flow and reaction sites. This simple, yet effective methodology ensures reproducibility and reliability in the sensor’s performance. To demonstrate the platform’s potential, glucose detection was selected as a model application. The portable CL system exhibited an excellent analytical performance, achieving a wide linear detection range from 10 µM to 1000 µM, with an impressive LOD of 8.68 µM. These results validate the platform’s suitability for the real-time monitoring of glucose levels, showcasing its potential for use in diverse applications. Rigorous testing confirmed the platform’s stability and repeatability under various conditions, further highlighting its robustness and reliability in real-world scenarios. The key innovation of this platform is the integration of deep learning, which significantly enhances the accuracy and efficiency of analyte detection. By analyzing CL images with advanced machine learning algorithms, the platform achieves high precision in quantifying analyte concentrations, reducing the errors associated with manual or traditional analysis. The deep learning integration also enables adaptability, allowing for the system to be trained for detecting various analytes beyond glucose. This work represents a significant step forward in making advanced diagnostic tools more accessible and affordable. By utilizing inexpensive materials, such as paper substrates, and harnessing the computational power of smartphones, the platform addresses the need for low-cost, portable diagnostic solutions. Its versatility extends beyond healthcare, offering potential applications in environmental monitoring, food safety, and other fields where rapid and accurate detection is critical. Overall, this deep learning-powered CL platform bridges the gap between high-end diagnostic tools and real-world accessibility. It democratizes biosensing technology by providing a flexible, scalable, and cost-effective solution, paving the way for next-generation biosensors. With its transformative potential, this work contributes significantly to advancing point-of-care diagnostics and fostering innovations across multiple domains requiring precise and reliable analyte detection.

## Figures and Tables

**Table 1:** Statistics of dataset prior to and following data augmentation.

Name of Sample Test Data	Dataset Before Augmentation			Dataset After Augmentation		
	Train Data	Test Data	Total Dataset	Train Data	Test Data	Total Dataset
**10 µM**	10	40	50	100	400	500
**50 µM**	10	40	50	100	400	500
**100 µM**	10	40	50	100	400	500
**200 µM**	10	40	50	100	400	500
**300 µM**	10	40	50	100	400	500
**400 µM**	10	40	50	100	400	500
**500 µM**	10	40	50	100	400	500
**600 µM**	10	40	50	100	400	500
**700 µM**	10	40	50	100	400	500
**800 µM**	10	40	50	100	400	500
**900 µM**	10	40	50	100	400	500
**1000 µM**	10	40	50	100	400	500
**Total**	**120**	**480**	**600**	**1200**	**4800**	**6000**

**Table 2:** Comparative analysis of various standardized deep learning models used.

Epoch/Model	KNN	Random Forest	InceptionV3	VGG16	RasNet-50	SVM
**10**	73	59	87	66	98	98
**15**	71	64	89	77	98	99
**20**	73	71	93	80	98	99
**25**	69	70	90	85	98	99
**30**	71	75	93	87	98	99
**35**	73	72	93	88	98	99
**40**	72	71	92	93	98	99
**Peak Accuracy**	**73**	**75**	**93**	**93**	**98**	**99**

**Table 3:** Validation of unknown glucose sample and prediction through Support Vector Machines.

Analyte Tested	Known Concentration (µM)	Lab Testing Result (µM)	Testing with WPµ-pad CL Device (µM)	Prediction Using SVM (µM)	Absolute Error Value
**Glucose**	150	151.34	148.85	149.059	0.941
	250	252.541	255.64	253.265	3.265
	450	456.321	444.026	445.621	4.379
	650	653.253	652.121	652.321	2.321
	850	853.984	843.789	845.967	4.033
	Average Mean Absolute Error Value				2.9878

**Table 4:** Comparative evaluation of chemiluminescent systems in biosensors for detecting diverse analytes.

Sr. No.	Sensing System/Materials Used	Application of Sensor	Linear Range	LOD	AI Algorithm Used	References
1.	HCC/HLG film	Glucose	0.01–50 nM and 50 nM–2.0 μM	9.0 pM	No	[64]
2.	Microplates	Glucose	0.1–2.5 mmol L^−1^	120 µmol L^−1^	No	[65]
3.	CCGTSs	Glucose	0.1–100 mM	0.0948 mM	No	[38]
4.	LFIA	cortisol	0.3–60 ng/mL	0.3 ng/mL	No	[66]
5.	Cu-MOXs	Dopamine	40–200 nM	10 nM	No	[67]
6.	MOFs	Glucose	0.2–100 mmol L^−1^	0.011 mM L^−1^	No	[68]
7.	Flow injection system	cholesterol	0.05–10 mM	1.5 μM	No	[69]
8.	HRP/COD/luminol/Alg	cholesterol	0.01–0.35 mM	7.2 µM	No	[70]
9.	Spectrometer	Cortisol	0.42 to 72.27 ng/mL	0.12 ng/mL	No	[71]
**10.**	**WPµ-pad**	**Glucose**	**10–1000 µM**	**8.68 µM**	**Yes**	**This Work**

HCC/HLG film: hollow structural calcium carbonate (HCC)/Lucigenin and GOx (HLG) film; CCGTSs: chemiluminescence cloth-based glucose test sensors; LFIA: lateral flow immunoassay; Cu-MOXs: copper-based metal–organic xerogels; MOFs: Metal–Organic Frameworks; HRP: Horseradish Peroxidase; COD: Cholesterol Oxidase; Alg: Alginate.

## Data Availability

The original contributions presented in this study are included in this article; further inquiries can be directed to the corresponding authors.

## References

[1] Wang C., Liu M., Wang Z., Li S., Deng Y., He N. (2021). Point-of-care diagnostics for infectious diseases: From methods to devices. Nano Today.

[2] Polat E.O., Cetin M.M., Tabak A.F., Bilget Güven E., Uysal B.Ö., Arsan T., Kabbani A., Hamed H., Gül S.B. (2022). Transducer Technologies for Biosensors and Their Wearable Applications. Biosensors.

[3] Si P., Huang Y., Wang T., Ma J. (2013). Nanomaterials for electrochemical non-enzymatic glucose biosensors. RSC Adv..

[4] Dhara K., Mahapatra D.R. (2018). Electrochemical nonenzymatic sensing of glucose using advanced nanomaterials. Microchim. Acta.

[5] Singh K., Agarwal T., Kumar U., Pal S., Runthala A., Pan T.M., Wu C.C. (2023). Optical biosensors for diabetes management: Advancing into stimuli-responsive sensing mechanisms. Smart Mater. Med..

[6] Rachim V.P., Chung W.-Y. (2019). Wearable-band type visible-near infrared optical biosensor for non-invasive blood glucose monitoring. Sens. Actuators B Chem..

[7] Kaur A., Kumar P., Gupta A., Sapra G. (2023). Piezoelectric Biosensors in Healthcare. Enzyme-Based Biosensors: Recent Advances and Applications in Healthcare.

[8] Zhao H., Guo X., Wang Y., Duan X., Qu H., Zhang H., Zhang D., Pang W. (2016). Microchip based electrochemical-piezoelectric integrated multi-mode sensing system for continuous glucose monitoring. Sens. Actuators B Chem..

[9] Zalke J.B., Pandey S.R., Narkhede N.P. (2024). An Ultrasonic Time-of-Flight Sensing System Based on Custom Hardware of Time to Digital Converter (TDC) for Measurement of Glucose Concentration. IEEE Sens. J..

[10] Sim J.Y., Ahn C.-G., Jeong E.-J., Kim B.K. (2018). In vivo Microscopic Photoacoustic Spectroscopy for Non-Invasive Glucose Monitoring Invulnerable to Skin Secretion Products. Sci. Rep..

[11] Mohankumar P., Ajayan J., Mohanraj T., Yasodharan R. (2021). Recent developments in biosensors for healthcare and biomedical applications: A review. Measurement.

[12] Huang C.-W., Lin C., Nguyen M.K., Hussain A., Bui X.-T., Ngo H.H. (2023). A review of biosensor for environmental monitoring: Principle, application, and corresponding achievement of sustainable development goals. Bioengineered.

[13] Lozano M.G., García Y.P., Gonzalez J.A.S., Bañuelos C.V.O., Escareño M.P.L., Balagurusamy N. (2019). Biosensors for Food Quality and Safety Monitoring: Fundamentals and Applications. Enzymes in Food Biotechnology.

[14] Heikenfeld J., Jajack A., Feldman B., Granger S.W., Gaitonde S., Begtrup G., Katchman B.A. (2019). Accessing analytes in biofluids for peripheral biochemical monitoring. Nat. Biotechnol..

[15] Jović M., Prim D., Saini E., Pfeifer M.E. (2022). Towards a Point-of-Care (POC) Diagnostic Platform for the Multiplex Electrochemiluminescent (ECL) Sensing of Mild Traumatic Brain Injury (mTBI) Biomarkers. Biosensors.

[16] Pradela-Filho L.A., Veloso W.B., Arantes I.V., Gongoni J.L., de Farias D.M., Araujo D.A., Paixão T.R. (2023). Paper-based analytical devices for point-of-need applications. Microchim. Acta.

[17] Xia S., Pan J., Dai D., Dai Z., Yang M., Yi C. (2023). Design of portable electrochemiluminescence sensing systems for point-of-care-testing applications. Chin. Chem. Lett..

[18] Ying X., Zhou L., Fu W., Wang Y., Su B. (2023). Electrochemiluminescence devices for point-of-care testing. Sens. Diagn..

[19] Chin C.D., Linder V., Sia S.K. (2012). Commercialization of microfluidic point-of-care diagnostic devices. Lab Chip.

[20] Haleem A., Javaid M., Singh R.P., Suman R., Rab S. (2021). Biosensors applications in medical field: A brief review. Sens. Int..

[21] Bhalla N., Jolly P., Formisano N., Estrela P. (2016). Introduction to biosensors. Essays Biochem..

[22] Bhatia D., Paul S., Acharjee T., Ramachairy S.S. (2024). Biosensors and their widespread impact on human health. Sens. Int..

[23] Heidt B., Siqueira W.F., Eersels K., Diliën H., van Grinsven B., Fujiwara R.T., Cleij T.J. (2020). Point of Care Diagnostics in Resource-Limited Settings: A Review of the Present and Future of PoC in Its Most Needed Environment. Biosensors.

[24] Senf B., Yeo W.-H., Kim J.-H. (2020). Recent Advances in Portable Biosensors for Biomarker Detection in Body Fluids. Biosensors.

[25] Srinivasan B., Tung S. (2015). Development and Applications of Portable Biosensors. SLAS Technol..

[26] Yang L., Li J. (2023). Recent Advances in Electrochemiluminescence Emitters for Biosensing and Imaging of Protein Biomarkers. Chemosensors.

[27] Qi H., Zhang C. (2020). Electrogenerated Chemiluminescence Biosensing. Anal. Chem..

[28] Yan Y., Shi P., Song W., Bi S. (2019). Chemiluminescence and Bioluminescence Imaging for Biosensing and Therapy: *In Vitro* and *In Vivo* Perspectives. Theranostics.

[29] Tzani M.A., Gioftsidou D.K., Kallitsakis M.G., Pliatsios N.V., Kalogiouri N.P., Angaridis P.A., Lykakis I.N., Terzidis M.A. (2021). Direct and Indirect Chemiluminescence: Reactions, Mechanisms and Challenges. Molecules.

[30] Das A., Paul P., Raj M., Sarkar A., De A., Banerjee T., Bhowmik R., Shaharyar M.A., Anand K., Biswas E. (2025). Chemiluminescence-based biosensor: From principle to its applications. Fundamentals of Biosensors in Healthcare.

[31] Luo M., Chen X., Zhou G., Xiang X., Chen L., Ji X., He Z. (2012). Chemiluminescence biosensors for DNA detection using graphene oxide and a horseradish peroxidase-mimicking DNAzyme. Chem. Commun..

[32] Heckenlaible N., Snyder S., Herchenbach P., Kava A., Henry C.S., Gross E.M. (2022). Comparison of Mobile Phone and CCD Cameras for Electrochemiluminescent Detection of Biogenic Amines. Sensors.

[33] Hao M., Liu N., Ma Z. (2013). A new luminol chemiluminescence sensor for glucose based on pH-dependent graphene oxide. Analyst.

[34] Park J.Y., Kricka L.J. (2014). Prospects for the commercialization of chemiluminescence-based point-of-care and on-site testing devices. Anal. Bioanal. Chem..

[35] Mirasoli M., Guardigli M., Michelini E., Roda A. (2014). Recent advancements in chemical luminescence-based lab-on-chip and microfluidic platforms for bioanalysis. J. Pharm. Biomed. Anal..

[36] Jang H., Noh H. (2015). Chemiluminescent detection of tear glucose on paper microfluidic devices. Macromol. Res..

[37] Ma Y., Sun X., Cai Z., Tu M., Wang Y., Ouyang Q., Yan X., Jing G., Yang G. (2024). Transformation gap from research findings to large-scale commercialized products in microfluidic field. Mater. Today Bio.

[38] Li H., Liu C., Wang D., Zhang C. (2017). Chemiluminescence cloth-based glucose test sensors (CCGTSs): A new class of chemiluminescence glucose sensors. Biosens. Bioelectron..

[39] Cui F., Yue Y., Zhang Y., Zhang Z., Zhou H.S. (2020). Advancing Biosensors with Machine Learning. ACS Sens..

[40] Bhaiyya M., Panigrahi D., Rewatkar P., Haick H. (2024). Role of Machine Learning Assisted Biosensors in Point-of-Care-Testing For Clinical Decisions. ACS Sens..

[41] Zalke J.B., Bhaiyya M.L., Jain P.A., Sakharkar D.N., Kalambe J., Narkhede N.P., Thakre M.B., Rotake D.R., Kulkarni M.B., Singh S.G. (2024). A Machine Learning Assisted Non-Enzymatic Electrochemical Biosensor to Detect Urea Based on Multi-Walled Carbon Nanotube Functionalized with Copper Oxide Micro-Flowers. Biosensors.

[42] Bhaiyya M., Rewatkar P., Pimpalkar A., Jain D., Srivastava S.K., Zalke J., Kalambe J., Balpande S., Kale P., Kalantri Y. (2024). Deep Learning-Assisted Smartphone-Based Electrochemiluminescence Visual Monitoring Biosensor: A Fully Integrated Portable Platform. Micromachines.

[43] Bhaiyya M.L., Srivastava S.K., Pattnaik P.K., Goel S. (2023). Closed-Bipolar Mini Electrochemiluminescence Sensor to Detect Various Biomarkers: A Machine Learning Approach. IEEE Trans. Instrum. Meas..

[44] Kumar A., Jain D., Bahuguna J., Bhaiyya M., Dubey S.K., Javed A., Goel S. (2023). Machine learning assisted and smartphone integrated homogeneous electrochemiluminescence biosensor platform for sample to answer detection of various human metabolites. Biosens. Bioelectron..

[45] Zhang Y., Cui Y., Sun M., Wang T., Liu T., Dai X., Zou P., Zhao Y., Wang X., Wang Y. (2022). Deep learning-assisted smartphone-based molecularly imprinted electrochemiluminescence detection sensing platform: Protable device and visual monitoring furosemide. Biosens. Bioelectron..

[46] Zhao P., Zhu W., Zheng M., Feng J. (2023). Deep Learning Enhanced Electrochemiluminescence Microscopy. Anal. Chem..

[47] Haghighi B., Tavakoli A., Bozorgzadeh S. (2016). Improved electrogenerated chemiluminescence of luminol by cobalt nanoparticles decorated multi-walled carbon nanotubes. J. Electroanal. Chem..

[48] Beigi S.M., Mesgari F., Hosseini M., Aghazadeh M., Ganjali M.R. (2019). An enhancement of luminol chemiluminescence by cobalt hydroxide decorated porous graphene and its application in glucose analysis. Anal. Methods.

[49] Zhang Z.-F., Cui H., Lai C.-Z., Liu L.-J. (2005). Gold Nanoparticle-Catalyzed Luminol Chemiluminescence and Its Analytical Applications. Anal. Chem..

[50] Spohn U., Preuschoff F., Blankenstein G., Janasek D., Kula M.R., Hacker A. (1995). Chemiluminometric enzyme sensors for flow-injection analysis. Anal. Chim. Acta.

[51] Kazemi M.S., Kazemi K., Yaghoobi M.A., Bazargan H. (2016). A hybrid method for estimating the process change point using support vector machine and fuzzy statistical clustering. Appl. Soft Comput..

[52] Ali L., Niamat A., Khan J.A., Golilarz N.A., Xingzhong X., Noor A., Nour R., Bukhari S.A. (2019). An Optimized Stacked Support Vector Machines Based Expert System for the Effective Prediction of Heart Failure. IEEE Access.

[53] Probst P., Wright M.N., Boulesteix A. (2019). Hyperparameters and tuning strategies for random forest. WIREs Data Min. Knowl. Discov..

[54] Speiser J.L., Miller M.E., Tooze J., Ip E. (2019). A comparison of random forest variable selection methods for classification prediction modeling. Expert Syst. Appl..

[55] Alam M.Z., Rahman M.S., Rahman M.S. (2019). A Random Forest based predictor for medical data classification using feature ranking. Inform. Med. Unlocked.

[56] Syriopoulos P.K., Kalampalikis N.G., Kotsiantis S.B., Vrahatis M.N. (2023). kNN Classification: A review. Ann. Math. Artif. Intell..

[57] Jabbar M.A., Deekshatulu B.L., Chandra P. (2013). Classification of Heart Disease Using K- Nearest Neighbor and Genetic Algorithm. Procedia Technol..

[58] Chen Y., Li L., Li W., Guo Q., Du Z., Xu Z. (2024). Deep learning. AI Computing Systems.

[59] Linkon A.H.M., Labib M.M., Hasan T., Hossain M., Jannat M.-E. (2021). Deep learning in prostate cancer diagnosis and Gleason grading in histopathology images: An extensive study. Inform. Med. Unlocked.

[60] Shoeibi A., Moridian P., Khodatars M., Ghassemi N., Jafari M., Alizadehsani R., Kong Y., Gorriz J.M., Ramírez J., Khosravi A. (2022). An overview of deep learning techniques for epileptic seizures detection and prediction based on neuroimaging modalities: Methods, challenges, and future works. Comput. Biol. Med..

[61] Sharma P., Nayak D.R., Balabantaray B.K., Tanveer M., Nayak R. (2024). A survey on cancer detection via convolutional neural networks: Current challenges and future directions. Neural Netw..

[62] Jing Y., Li C., Du T., Jiang T., Sun H., Yang J., Shi L., Gao M., Grzegorzek M., Li X. (2023). A comprehensive survey of intestine histopathological image analysis using machine vision approaches. Comput. Biol. Med..

[63] Oğuz A., Ertuğrul Ö.F. (2023). Introduction to deep learning and diagnosis in medicine. Diagnostic Biomedical Signal and Image Processing Applications with Deep Learning Methods.

[64] Wang C., Zhou C., Long Y., Cai H., Yin C., Yang Q., Xiao D. (2016). An enhanced chemiluminescence bioplatform by confining glucose oxidase in hollow calcium carbonate particles. Sci. Rep..

[65] Calabria D., Pace A., Lazzarini E., Trozzi I., Zangheri M., Guardigli M., Pieraccini S., Masiero S., Mirasoli M. (2023). Smartphone-Based Chemiluminescence Glucose Biosensor Employing a Peroxidase-Mimicking, Guanosine-Based Self-Assembled Hydrogel. Biosensors.

[66] Zangheri M., Cevenini L., Anfossi L., Baggiani C., Simoni P., Di Nardo F., Roda A. (2015). A simple and compact smartphone accessory for quantitative chemiluminescence-based lateral flow immunoassay for salivary cortisol detection. Biosens. Bioelectron..

[67] Zhang L., Hou Y., Lv C., Liu W., Zhang Z., Peng X. (2020). Copper-based metal–organic xerogels on paper for chemiluminescence detection of dopamine. Anal. Methods.

[68] Song M., Shi F., Zhang R., Wang X., Sun X., Li Y., Ren X., Ma H., Wei Q. (2022). Long-lasting chemiluminescence bioassays for glucose enabled by a MOFs-in-hydrogel hybrid platform. Sens. Diagn..

[69] Xu S., Wang Y., Zhou D., Kuang M., Fang D., Yang W., Wei S., Ma L. (2016). A novel chemiluminescence sensor for sensitive detection of cholesterol based on the peroxidase-like activity of copper nanoclusters. Sci. Rep..

[70] Ning Y., Lu F., Liu Y., Yang S., Wang F., Ji X., He Z. (2021). Glow-type chemiluminescent hydrogels for point-of-care testing (POCT) of cholesterol. Analyst.

[71] Fu H.J., Yuan L.P., Shen Y.D., Liu Y.X., Liu B., Zhang S.W., Xie Z.X., Lei H.T., Sun Y.M., Xu Z.L. (2018). A full-automated magnetic particle-based chemiluminescence immunoassay for rapid detection of cortisol in milk. Anal. Chim. Acta.

